# Detection of Alzheimer’s disease using Otsu thresholding with tunicate swarm algorithm and deep belief network

**DOI:** 10.3389/fphys.2024.1380459

**Published:** 2024-07-09

**Authors:** Praveena Ganesan, G. P. Ramesh, Przemysław Falkowski-Gilski, Bożena Falkowska-Gilska

**Affiliations:** ^1^ Department of Electronics and Communication Engineering, St. Peter’s Institute of Higher Education and Research, Chennai, India; ^2^ Faculty of Electronics, Telecommunications and Informatics, Gdansk University of Technology, Gdansk, Poland; ^3^ Specialist Diabetes Outpatient Clinic, Olsztyn, Poland

**Keywords:** Alzheimer’s disease detection, classification accuracy, deep belief network, magnetic resonance imaging, Otsu thresholding, tunicate swarm algorithm

## Abstract

**Introduction:** Alzheimer’s Disease (AD) is a degenerative brain disorder characterized by cognitive and memory dysfunctions. The early detection of AD is necessary to reduce the mortality rate through slowing down its progression. The prevention and detection of AD is the emerging research topic for many researchers. The structural Magnetic Resonance Imaging (sMRI) is an extensively used imaging technique in detection of AD, because it efficiently reflects the brain variations.

**Methods:** Machine learning and deep learning models are widely applied on sMRI images for AD detection to accelerate the diagnosis process and to assist clinicians for timely treatment. In this article, an effective automated framework is implemented for early detection of AD. At first, the Region of Interest (RoI) is segmented from the acquired sMRI images by employing Otsu thresholding method with Tunicate Swarm Algorithm (TSA). The TSA finds the optimal segmentation threshold value for Otsu thresholding method. Then, the vectors are extracted from the RoI by applying Local Binary Pattern (LBP) and Local Directional Pattern variance (LDPv) descriptors. At last, the extracted vectors are passed to Deep Belief Networks (DBN) for image classification.

**Results and Discussion:** The proposed framework achieves supreme classification accuracy of 99.80% and 99.92% on the Alzheimer’s Disease Neuroimaging Initiative (ADNI) and Australian Imaging, Biomarker and Lifestyle flagship work of ageing (AIBL) datasets, which is higher than the conventional detection models.

## 1 Introduction

The brain is the most complex and significant organ of the human being with numerous crucial functions like memory, imagination, decision-making, thinking, problem solving, and idea formation (Basaia et al., 2019; Li et al., 2018). AD is the common degenerative brain disorder; approximately 48 million people suffer from this disease and other types of dementias. AD occurs due to the abnormal chemical reactions, head injuries, genetic factors, and environmental factors (Sathiyamoorthi et al., 2021; Chui et al., 2022). The main symptoms of AD are behavior disruption, communication problems, recognition problems, cognition problems, and memory loss. AD leads to the death of brain cells, which causes cognitive power, thinking, and loss of memory (Vaithinathan and Parthiban, 2019). The speed of AD progression differs from patient to patient, but it has a low diagnosis rate. The behavioral disturbance caused by the AD impairs the social functioning of patients (Zhang et al., 2019). The AD generally affects elderly people and even leads to death, when it is not treated and detected at an early stage. Therefore, it is essential to detect AD at an early stage to reduce its progression and decrease the mortality rate (Yue et al., 2019; Ghazi et al., 2021; Ning et al., 2021).

In recent decades, several brain-imaging techniques such as computed tomography, sMRI, positron emission tomography, functional MRI, etc., are used for early diagnosis of AD (Puente-Castro et al., 2020; Chelladurai et al., 2023). Compared to other brain-imaging techniques, sMRI images provide functional information and complementary structural information about the abnormal brain regions (Liu et al., 2021). The simplicity and quickness are considered as two of its numerous advantages of Otsu’s approach. The best threshold value to distinguish the foreground from background portions of the processed image which can be automatically determined, eliminating the need for prior knowledge of the image. For automating the AD detection, several machine-learning models such as decision tree, Support Vector Machine (SVM), k-nearest neighbors, XGBoost, etc., are employed, but the conventional models are prone to outliers and overfitting risks (Alqahtani et al., 2023; Ghosh et al., 2023). On the other hand, the deep-learning models have got more attention among researchers and also brought dramatic advancements and improvements in medical imaging, computer vision, image processing, and pattern recognition applications (Venugopalan et al., 2021; Qu et al., 2023). Deep Belief Networks (DBN) provide certain advantages over traditional neural networks by utilizing supervised learning and probabilistic modeling. It can process enormous quantities of data and use hidden units to quickly identify underlying correlations through fast training. The above-stated information’s motivates to detect the AD with minimum execution time by employing DBN models. The main contributions of the present article are denoted as follows:

• TSA is integrated with the Otsu thresholding method for precise RoI segmentation in the acquired sMRI images. The main motivation behind the Otsu thresholding method is to maximize the between class variance for determining the optimal threshold value, but it consumes more time. Therefore, in the conventional Otsu thresholding method, the TSA is employed to select the optimal threshold value for accurate RoI segmentation with minimal execution time.
• Then combined the LBP and LDPv descriptors for extracting texture feature vectors from the segmented RoI. Because of high discriminative power, the extraction of global feature vectors from sMRI images is computationally effective and robust.
• The extracted global feature vectors are passed into the DBN model for classifying four classes in the AIBL dataset and three classes in the ADNI dataset. In comparison to other classification models, the suggested DBN model efficiently captures non-linear feature vectors from the extracted feature vectors, which improves its ability in discriminating different classes. The performance of the Otsu-TSA method and DBN model are validated in terms of Jaccard Similarity Coefficient (JSC), Dice Similarity Coefficient (DSC), Pixel Accuracy (PA), specificity, execution time, classification accuracy, and sensitivity.

The structure of this research is organized as follows; The recent research papers on the topic AD detection are reviewed in Section 2. The details about the proposed methods (Otsu-TSA method and DBN model), and its mathematical equations are described in Section 3; The results analysis and comparisons of the proposed method are demonstrated in Section 4; finally, the conclusion of this research is stated in Section 5.

## 2 Related works

Xiao et al. (2017) performed scaling correction, intensity based non-uniformity correction, B1 non-uniformity correction, and gradient warping for enhancing the quality of sMRI images. Further, the textural and morphometric vectors were extracted from the pre-processed sMRI images. Then, the discriminative optimal vectors were selected by applying a recursive feature elimination technique, which were further passed to the SVM classifier for AD detection. The developed framework was only appropriate in the binary-class classification.

Ebrahimi-Ghahnavieh et al. (2019) performed transfer learning on MRI images for precise detection of AD. In this study, various pre-trained CNNs were applied on the datasets with multi-view and single-view modes. Initially, the vectors were extracted from the MRI images by employing CNN model, and the extracted vectors were passed to the Recurrent Neural Network (RNN) for image classification. The RNN faces concerns like gradient vanishing or exploding, and the computation of the neural network was too slow in image classification.

Lian et al. (2020) presented a hierarchical based Fully-CNN model for determining the discriminative local regions and patches in the MRI images for early detection of AD. Additionally, Bi et al. (2020) implemented a stacked CNN model for AD diagnosis. The presented CNN models achieved significant performance in AD detection on the ADNI datasets, but showed high time complexity. Furthermore, Hu et al. (2020) used the GAN model for AD detection by addressing class imbalance problems. In the context of image classification, training the GAN model was time consuming and computationally intensive.

Zhu et al. (2021) developed a Dual Attention Multi Instance Deep Learning (DA-MIDL) model for AD and Mild Cognitive Impairment (MCI) detection in the sMRI images. The developed DA-MIDL model had three major processes; (i) incorporate numerous spatial attention blocks in the Patch-Net model to extract discriminative vectors, (ii) perform pooling operation to balance every sMRI patch, and (iii) use a global attention aware classification model for making classification decisions. The results demonstrate that the developed DA-MIDL model achieved better classification performance by precisely identifying the pathological locations. The generalizability and classification accuracy were better than traditional methods. However, overfitting and lack of interpretability were the major concerns in the DA-MIDL model.

Mehmood et al. (2021) performed the tissue segmentation and classification using Layer Wise Transfer Learning (LWTL) for early detection of AD. Here, the LWTL was carried-out in the Visual Geometry Group (VGG) model for segregating Normal Controls (NC), AD, Late MCI (LMCI), and Early MCI (EMCI).

Ashraf et al. (2021) used 2nd and 3rd generation neural networks for AD classification. Additionally, several image augmentation techniques were utilized for improving feature extraction in the MRI images. Around 13 deep learning models were fine-tuned and trained on the ImageNet datasets. Among the trained models, the DenseNet model was more impressive in the AD classification than other models. Janghel and Rathore (2021) initially resized the collected images, and further, the VGG-16 model was employed for discriminative feature extraction. Lastly, the image classification was carried out utilizing decision tree, k-means clustering, and SVM. The results indicated that the developed model had better classification accuracy, but the execution time was high on the ADNI datasets.

Kang et al. (2021) presented an effective ensemble learning system based on CNN model for precise AD detection. The presented system included two phases: a majority voting was carried out in the first phase for disease prediction, and in the second phase, ResNet50 and VGG-16 models were integrated for constructing an ensemble learning classifier. Here, two transfer-learning strategies (task adaptation transfer and domain transfer) were utilized to manage the biomedical classification problems. The results represented that the presented ensemble learning system achieved better classification results than the single classifiers, but it exhibited high time complexity.

Fan et al. (2021) implemented a U-Net model for precise classification of AD. The U-Net model was effective in both image segmentation and classification tasks, while the skip-connections in the U-Net model efficiently improved the classification performance. While working with smaller datasets, the skip-connections in the U-Net model were prone to overfitting problems.

Liu et al. (2021) presented an effective framework for AD classification based on the CNNs. Initially, the brain images were portioned into dissimilar regions, and then the K-means clustering technique was employed for grouping the similar patches from every region. Finally, the features were learned from the grouped regions by employing the DenseNet model for image classification. In the DenseNet model, excessive parameters that led to overfitting problems were processed.

Naz et al. (2022) used convolutional networks with freeze-features for classifying AD, NC, and MCI in MRI scans. The presented framework had limited computational complexity and underwent fast training in relation to the conventional methods. AlSaeed and Omar (2022) utilized the ResNet50 and stacked Convolutional Neural Network (CNN) models for effective AD detection. The efficacy of the presented model was compared with traditional methods by using various evaluation measures, including accuracy. Here, the performance of the convolutional networks completely depends on the image quality.

Alhassan (2022) initially performed image pre-processing using skull stripping, linear registration, and geometry correction techniques. Then, the region segmentation was carried out by integrating the Otsu thresholding method with the Enhanced Fuzzy Elephant Herding Optimization (EFEHO) algorithm. The EFEHO algorithm quickly selected the optimal threshold value in the Otsu thresholding method for precise segmentation. Finally, the DA-MIDL model was implemented for AD classification, and the model’s efficacy was analyzed in the light of specificity, accuracy, and sensitivity. As a result, the DA-MIDL model had problems such as lack of interpretability and overfitting.

Rani Kaka and Prasad (2022) used region growing and adaptive histogram equalization techniques for removing the skull region and improving contrast of the MRI images. Then, the interested regions were segmented using the fuzzy c means technique, and further, the vectors were extracted using a local directional pattern and Gabor features. Finally, the feature selection and classification were carried-out by implementing a correlation/ensemble-based feature selection technique and a Multi-class SVM (MSVM) classifier. However, the MSVM classifier was inappropriate in the larger datasets when the target classes were overlapped.

Yu et al. (2022) introduced a multi-directional perception based Generative Adversarial Network (GAN) model for determining the severity of AD. The presented model efficiently captures salient global features and learns class discriminative maps for dissimilar classes by integrating L1 penalty, cycle consistency loss, classification loss, adversarial loss, and multi-directional mapping process in the conventional GAN model. Wang et al. (2022) developed a consistent perception-based GAN for precise segmentation of stroke lesions. The suggested method has been used for solving the imbalance problems, but it was time consuming and computationally intensive.

Shukla et al. (2024a) has demonstrated Structural biomarker-based AD detection via ensemble learning approaches. This work used ensemble learning and standard machine learning models to detect AD and its subtypes. The relative impact scores of the different AD cortical and subcortical areas and their subtypes were also determined. Binary and multiclass classification are the two levels used in the experimental investigation. The parahippocampal and entorhinal areas of the right hemisphere were found to be the most significant in the cortical-subcortical study. In the left hemisphere, the inferior temporal and isthmus cingulate regions also had a notable impact. However, more study remains required to develop sustainable methods for detecting AD and its stages.

Shukla et al. (2024b) analyzed a subcortical structure in AD using ensemble learning approaches. The combination of MRI and PET modalities is shown in this work, which could boost result visualization. Second, we analyzed the various subcortical structures using the Ensemble Model (EM) and further machine learning (ML) techniques to ascertain which region is more crucial for AD identification than other subtypes. The findings showed that the best regions for identifying AD, Mild Cognitive Impairment (MCI), And Cognitive Normal (CN) were the hippocampal, neuroregion, and the amygdala in the left and right hemispheres. Still, further data may be required to properly categorize AD and its stages.

Shukla et al. (2023) has presented AD detection from fused PET and MRI modalities with the help of an ensemble classifier. The multimodal approach is used in this paper to extensively explore the prediction of AD and its stages. Through the affine registration method, two modalities (MRI and PET) can be combined with the use of an automatic pipeline method called Free Surfer. The entire method makes use of pixel-level fusion. Following that, feature extraction is performed once more on both the fused and non-fused results. the traits that were taken from several cortical and subcortical brain regions, such as the putamen, amygdala, and hippocampus. The optimal qualities for categorization are retained after superimposing the fused features of both modalities and eliminating extraneous aspects. The promise of image- and feature-level fusion approaches as features for ensemble classification models was demonstrated, and these models exhibit sustained performance in Binary and Multi Class classification. On the other hand, accuracy for Multi Class is also respectable, however it can still be improved upon when compared to the Binary findings. Table 1 provides the tabulation form for the summary of AD classification papers focused on ADNI and AIBL datasets.

**TABLE 1:** Summary of Literature review of AD classification using ADNI and AIBL dataset.

Year	Author	Methodology and advantages	Limitations
2017	Xiao et al.	Support Vector Machine (SVM) enhanced the quality of sMRI images	Only appropriate in the binary-class classification
2019	Ebrahimi-Ghahnavieh et al.	Recurrent Neural Network (RNN) provided the precise detection.	Computation of the neural network was too slow in image classification
2020	Lian et al.	Hierarchical based Fully-CNN model determining the discriminative local regions and patches in the MRI images	Required large datasets
2020	Bi et al.	Stacked CNN achieved significant performance in AD detection	high time complexity and time consuming
2020	Hu et al.	Generative Adversarial Network (GAN) model solved the imbalance problems	Time consuming and computationally intensive
2021	Zhu et al.	Dual Attention Multi Instance Deep Learning (DA-MIDL) model achieved better classification performance by precisely identifying the pathological locations	Overfitting and lack of interpretability were the major concerns
2021	Mehmood et al.	Tissue segmentation and classification using Layer Wise Transfer Learning (LWTL) for precise classification	Required proper selection
2021	Ashraf et al.	2nd and 3rd generation neural networks for AD classification was more impressive in the AD classification than other models	High time complexity
2021	Janghel and Rathore	decision tree, k-means clustering, and SVM achieves better classification accuracy	Execution time was high on the ADNI datasets
2021	Kang et al.	Effective ensemble learning system based on CNN model achieved better classification results	High time complexity
2021	Fan et al.	U-Net model for precise classification of AD efficiently improved the classification performance	Skip-links in the U-NET model are prone to overfitting problems when working with small datasets.
2021	Liu et al.	The features were learned from the grouped regions by employing the DenseNet model for image classification	Excessive parameters that led to overfitting problems
2022	Naz et al.	Convolutional networks with freeze-features for classifying AD, NC, and MCI in MRI scans	Limited computational complexity
2022	AlSaeed and Omar	ResNet50 and stacked Convolutional Neural Network (CNN) for efficient classification	Performance of the convolutional networks completely depends on the image quality
2022	Alhassan et al.	Dual Attention Multi Instance Deep Learning (DA-MIDL) model obtains high model’s efficacy	lack of interpretability and overfitting
2022	Rani Kaka and Prasad	Region growing and adaptive histogram equalization techniques improved contrast of the MRI images	MSVM classifier was inappropriate in the larger datasets when the target classes were overlapped.
2022	Yu et al.	Multi-directional perception based Generative Adversarial Network (GAN) efficiently captures salient global features and learns class discriminative maps for dissimilar classes	GANs were especially difficult to train.
2022	Wang et al.	Consistent perception-based GAN solved the imbalance problems	Time consuming and computationally intensive
2024	Shukla A et al.	Ensemble Learning approaches for Structural biomarker-based AD detection	However, more study remains required to develop sustainable methods for detecting AD and its stages.
2024	Shukla A et al.	Ensemble Learning approaches to analyze a subcortical structure in AD.	Still, further data may be required to properly categorize AD and its stages.
2023	Shukla A et al.	Ensemble Learning approaches to extensively explore the prediction of AD and its stages.	On the other hand, accuracy for Multi Class is also respectable, however it can still be improved upon when compared to the Binary findings.

By reviewing the previous studies, deep learning models gained more attention among the researchers for AD detection. In the image segmentation and classification tasks, the majority of the deep learning models often lack interpretability. Furthermore, brain image data results in higher dimensional feature spaces. Effective feature extraction techniques are required to improve the performance of classification models and to avoid the curse of dimensionality problems. In order to overcome the aforementioned issues, an effective automated framework is proposed in this article for precise detection of AD in the sMRI images. The automated framework includes Otsu-TSA method, LDPv descriptor, LBP descriptor, and DBN model for AD detection.

## 3 Methodology

**FIGURE 1:**
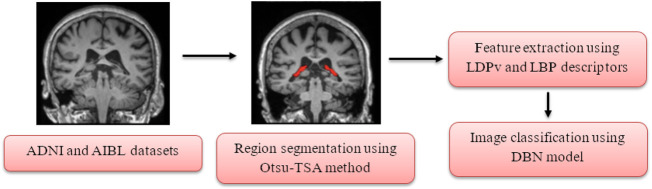
Working process of the proposed AD detection framework.

In the present scenario, the early detection of AD increases the survival rate of the patient. The deep learning and machine learning models on the sMRI images are extensively utilized in the detection of AD for accelerating the process of diagnosis and assisting clinicians in timely treatment. In this article, the proposed framework comprises of four steps in AD detection which include, dataset description: ADNI and AIBL datasets, region segmentation: Otsu-TSA method, feature extraction: LDPv and LBP descriptors, and classification: DBN model. The working process of the proposed AD detection framework is shown in Figure 1.

### 3.1 Dataset description

**FIGURE 2:**
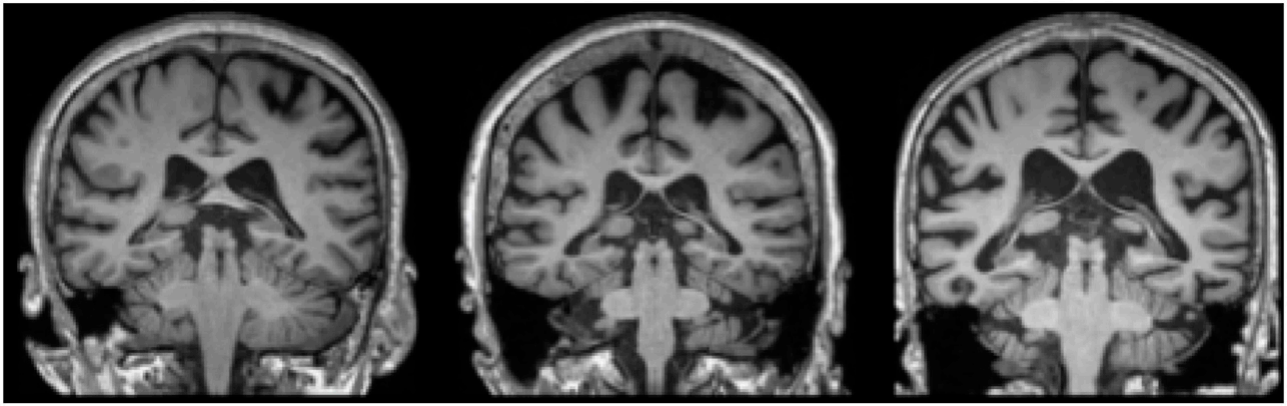
Sample acquired sMRI images from ADNI and AIBL datasets.

The performance of the proposed AD detection framework is evaluated on two benchmark-balanced datasets namely ADNI and AIBL. At first, the ADNI dataset has 1,662 sMRI images, where the subjects are classified into three types: NC, MCI, and AD based on the clinical dementia ratings and Mini-Mental State Examination (MMSE) scores. The clinical and demographic information of the ADNI and AIBL datasets are described in Table 2 (Yue et al., 2019). Secondly, the AIBL dataset has sMRI images (Zhu et al. (2021), which are recorded from 496 subjects, out of which 307 subjects belong to NC, 79 subjects are AD, 93 subjects are stable MCI (sMCI), and 17 subjects are progressive MCI (pMCI) (Alhassan, 2022). The sample acquired sMRI images from ADNI and AIBL datasets are specified in Figure 2.

**TABLE 2:** Clinical and demographic information about the ADNI and AIBL dataset.

Datasets	Classes	Number of images	Subjects Male/Female)	Age	MMSE scores
ADNI	AD	335	180/156	78.56 ± 5.34	23.84 ± 2.10
	MCI	542	349/193	78.86 ± 5.35	26.56 ± 2.63
	NC	785	369/416	74.63 ± 3.69	29.07 ± 1.32
AIBL	AD	335	33/46	73.34 ± 7.77	20.42 ± 5.46
	MCI	542	9/8	75.29 ± 6.16	26.24 ± 2.04
	NC	785	134/173	73.12 ± 6.19	28.77 ± 1.25

### 3.2 Region segmentation

After collecting the sMRI images, the RoI segmentation is carried-out using the Otsu-TSA method. The conventional Otsu thresholding method determines the maximum separability between the classes (3 classes in the ADNI dataset and 4 classes in the AIBL dataset) (Feng et al., 2017). The conventional Otsu thresholding method improves the segmentation results by comparing the average pixel value of the sMRI images with the selected pixel value. This segmentation method initially partitioned the sMRI images into two regions: dark region $T_{1}$ and light region $T_{0}$ (Goh et al., 2018; Tan et al., 2021). The mathematical representation of the dark region $T_{1}$ and light region $T_{0}$ are provided in Eqs 1, 2.

$${\;T}_{1}=\left\{t,t+1,\ldots .,l-1,l\right\}$$ (1)

$$T_{0}=\left\{0,1,\ldots .,t\right\}$$ (2)

The term t and l are indicated as threshold value and histogram bins, respectively. It is essential to select the optimal threshold value for distinguishing the overlapped classes. Generally, in the conventional Otsu thresholding method, the optimal threshold value is selected by reducing the variance of the weight based on the probability of distinct groups $d\left(i\right)$ , and it is specified in Eq. 3.

$$d\left(i\right)=\frac{number\;\left\{\left.\left(r,c\right)\right|image\left(r,c\right)=i\right\}}{\left(r,c\right)}$$ (3)

where, $\left(r,c\right)$ is represented as the rows and columns in the acquired sMRI images. The elements like variance $\sigma_{b}^{2}\left(t\right)$ and $\sigma_{f}^{2}\left(t\right)$ , mean ${\;\mu }_{b}\left(t\right)$ and ${\;\mu }_{f}\left(t\right)$ , and weight $w_{b}\left(t\right)$ and $w_{f}\left(t\right)$ of the light and dark regions ${\;T}_{0}$ and ${\;T}_{1}$ in the sMRI images are determined using Eqs 4, 5, where, background and foreground regions are represented as b and f .

$$w_{b}\left(t\right)=\sum_{i=1}^{t}d\left(i\right),{\;\mu }_{b}\left(t\right)=\frac{\sum_{i=1}^{t}i\times d\left(i\right)}{w_{b}\left(t\right)}\text{ and }\sigma_{b}^{2}\left(t\right)=\frac{\sum_{i=1}^{t}{\left(i-\mu_{b}\left(t\right)\right)}^{2}\times d\left(i\right)}{w_{b}\left(t\right)}$$ (4)

$$w_{f}\left(t\right)=\sum_{i=t+1}^{l}d\left(i\right),{\;\mu }_{f}\left(t\right)=\frac{\sum_{i=t+1}^{l}i\times d\left(i\right)}{w_{f}\left(t\right)}\text{ and }\sigma_{f}^{2}\left(t\right)=\frac{\sum_{i=t+1}^{l}{\left(i-\mu_{f}\left(t\right)\right)}^{2}\times d\left(i\right)}{w_{f}\left(t\right)}$$ (5)

The optimal threshold value is determined with low class variance $\sigma_{w}^{2}$ in the conventional Otsu thresholding method, but it is a time-consuming process. The low-class variance $\sigma_{w}^{2}$ is mathematically denoted in Eq. 6. Therefore, TSA is incorporated with the conventional Otsu thresholding method for finding the optimal threshold value with minimal execution time.

$$\sigma_{w}^{2}=w_{b}\left(t\right)\times \sigma_{b}^{2}\left(t\right)+w_{f}\left(t\right)\times \sigma_{f}^{2}\left(t\right)$$ (6)

TSA has drawn a lot of attention because of its straightforward design, small number of parameters, quick iteration, and high search functionality. TSA has been used by researchers to tackle optimization issues in a variety of domains, and they have discovered that TSA is rather effective in solving several real-world application issues. The TSA is one of the recent and effective optimization algorithms, which mimics the foraging behavior of the tunicates in ocean (Kaur et al., 2020). The TSA works based on two constraints: (i) it follows the positions of the qualified agents and, (ii) prevents the conflicts between the exploitation and exploration agents. The positions of the new agents are computed according to Eqs 7–9 for preventing the inter-agent conflicts (Houssein et al., 2021; Arabali et al., 2022).

$$\vec{A}=\frac{\vec{G}}{\vec{U}}$$ (7)

where,

$$\vec{G}=c_{2}+c_{3}-\vec{F}$$ (8)

$$\vec{F}=c_{1}\times \vec{F}$$ (9)

where, the random numbers are represented as $c_{1},c_{2}$ and $c_{3}$ , $\vec{A}$ is denoted as the vectors of the agent’s new positions, $\vec{F}$ is stated as the water flow, and the social forces between the agents are stored in a new vector $\vec{U}$ , which is mathematically expressed in Eq. 10.

$$\vec{U}=\left[O_{\min}+c_{1}\times O_{\max}-O_{\min}\right]$$ (10)

where, $O_{\min}=1$ and $O_{\max}=4$ are represented as the first and second subordinates, respectively, where these subordinates indicate the speed of the social interactions. In this optimization algorithm, it is essential to follow the present best agents to reach the optimal solution. Eq. 11 is used for computing the best positions of the best search agents.

$$\left.\vec{OD}\left.=\right|X_{best}-r_{rand}\times \vec{O_{o}\left(x\right)}\right|$$ (11)

where, the best position is represented as $X_{best}$ , the stochastic value is denoted as $r_{rand}$ , which ranges between 0 to 1, and the tunicate’s position during iteration is indicated as $\vec{O_{o}\left(x\right)}$ . The term $\vec{OD}$ denotes the length between the optimal agent and the food origin. Further, Eq. 12 is used to bring the search agents close to the best agents.

$$\vec{O_{o}\left(x\right)}=\left\{\begin{array}{c} X_{best}+A\times \vec{OD}\;if\;r_{rand}\geq 0.5\\ X_{best}-A\times \vec{OD}\;if\;r_{rand}<0.5 \end{array}\right\}$$ (12)

where, A is denoted as the agent’s new positions. The positions of the present agents are updated based on the positions of neighboring agents in order to model the tunicate’s swarming behaviour, as mentioned in Eq. 13. Here, finding the optimal threshold value of the Otsu thresholding method is the objective function, which is equal to 0.4. The TSA method terminates after reaching the maximum iteration number.

$$\vec{O_{o}\left(x+1\right)}=\frac{\vec{O_{o}\left(x\right)}+\vec{O_{o}\left(x+1\right)}}{2+c_{1}}$$ (13)

**FIGURE 3:**
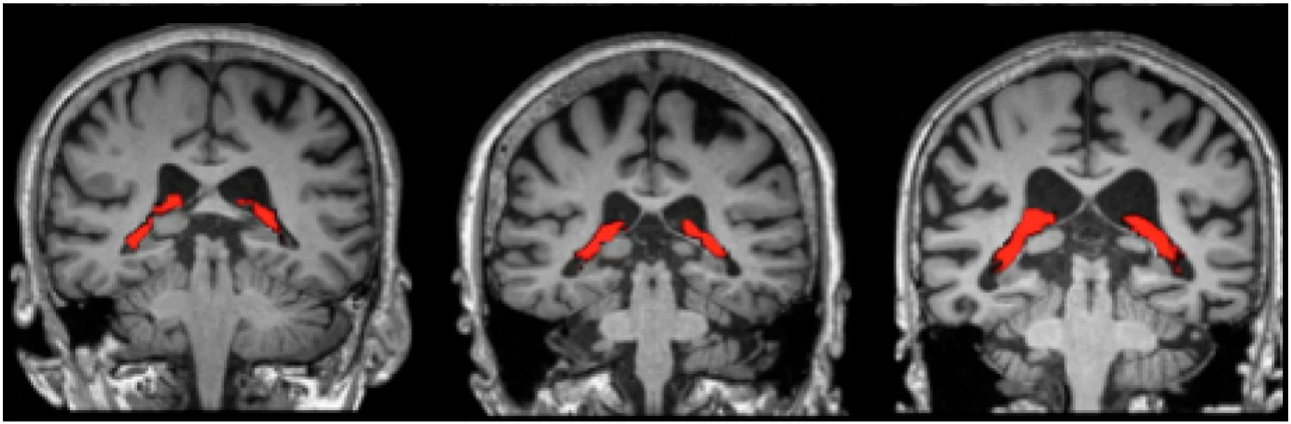
Sample segmented sMRI images.

The parameters fixed in the TSA method are represented as follows: number of population is 30, maximum iteration number is 100, $c_{1}$ is 0.7, $c_{2}$ is 0.3, and $c_{3}$ is 0.5. After the RoI segmentation, the feature extraction is carried out utilizing the LDPv and LBP descriptors. The sample segmented sMRI images are shown in Figure 3.

### 3.3 Feature extraction

After the RoI segmentation, the images are converted into vectors by employing the two global descriptors LDPv and LBP. At first, the LDPv descriptor extracts the vectors from the sMRI images by encoding the directional patterns and contrast information (Jabid et al., 2010). In this descriptor, a variance σ is included in the LDP as an adaptive weight for adjusting the LDP codes τ , while generating the histograms (Kabir et al., 2010; Kabir et al., 2012). The mathematical expressions of the LDPv descriptor are presented in Eqs 14–16, where M and N state the size of the segmented sMRI images. Approximately, 3,722 and 2,982 texture vectors are extracted from the ADNI and AIBL datasets by employing a LDPv descriptor.

$$LDPv\left(\tau \right)=\sum_{r=1}^{M}\sum_{c=1}^{N}w\left(LDP\left(r,c\right),\tau \right)$$ (14)

$$w\left(LDP\left(r,c\right),\tau \right)=\left\{\begin{array}{cc} \sigma \left(LDP\left(r,c\right)\right) & LDP\left(r,c\right)=\tau \\ 0 & otherwise \end{array}\right\}$$ (15)

$$\sigma \left(LDP\left(r,c\right)\right)=\frac{1}{8}{\sum_{i=0}^{7}\left(m_{i}-\bar{m}\right)}^{2}$$ (16)

**FIGURE 4:**
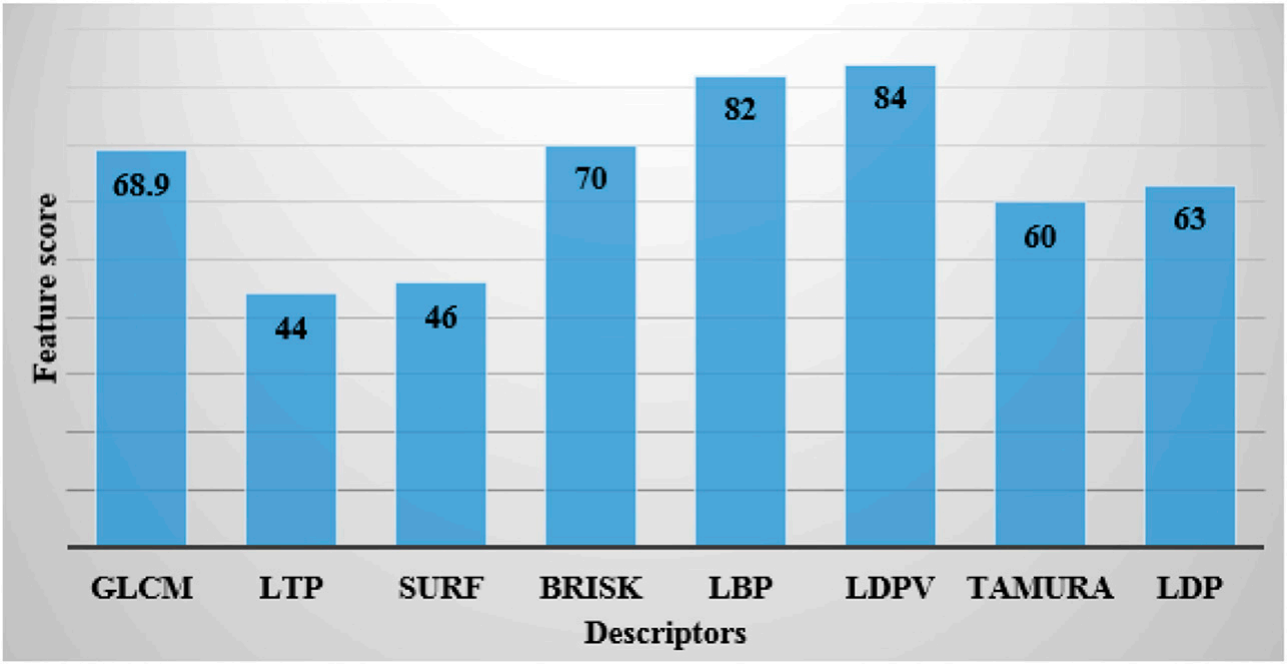
Calculation of feature importance scores.

The mean of all directional responses $m_{i}$ is represented as $\bar{m}$ , and it is computed in a position of $\left(r,c\right)$ , where *r* represents rows and *c* represents columns. Secondly, the LBP is one of the most efficient texture descriptors in medical image processing, which thresholds the neighborhood pixels on the basis of the current pixel value. The LBP descriptor superiorly captures the grey-scale contrast and local spatial patterns in the sMRI images (Kaplan et al., 2020). In the LBP descriptor, the neighborhood set is defined by the radius Ra and number of pixels P . Here, the centered pixel is represented as $g_{c}$ . The general formula of the LBP descriptor is mentioned in Eqs 17, 18.

$${LBP}_{P,Ra}=\sum_{p=0}^{p-1}s\left(g_{p}-{\;g}_{c}\right)2^{p}$$ (17)

where,

$$s\left(x\right)=\left\{\begin{array}{cc} 1 & x\geq 0\\ 0 & x<0 \end{array}\right\}$$ (18)

where, P is fixed as 8 and the radius Ra is 1. The function $s\left(x\right)$ is equal to 1 when the difference is over the threshold value 0 and otherwise, the function $s\left(x\right)$ is equal to 0 (Tuncer et al., 2020; Pan et al., 2021). A binomial factor $2^{p}$ is assigned in the LBP descriptor for the spatial structure of the local texture. The LBP descriptor extracts 2,820 and 2,207 vectors from the ADNI and AIBL datasets, respectively. The obtained vectors are passed as input to the DBN model for image classification. In this article, these two descriptors are employed by computing feature importance scores on the sMRI images, which is graphically shown in Figure 4.

### 3.4 Classification

The vectors extracted by using the LDPv and LBP descriptors are combined by performing feature level fusion. Around 6,542 and 5,189 vectors are extracted from the ADNI and AIBL datasets which are passed to the DBN model for image classification. The DBN is a probability generation model, which is trained in a greedy manner and stacked by Restricted Boltzmann Machine (RBM) (Sabar et al., 2020; Wang et al., 2020). In the DBN model, the previous layer’s output is passed as the input to the next layer. The hierarchical learning of the DBN model is inspired from the structure of the human brain (Hassan et al., 2019). Every layer in the DBN model is regarded as a logistic regression model.

**FIGURE 5:**
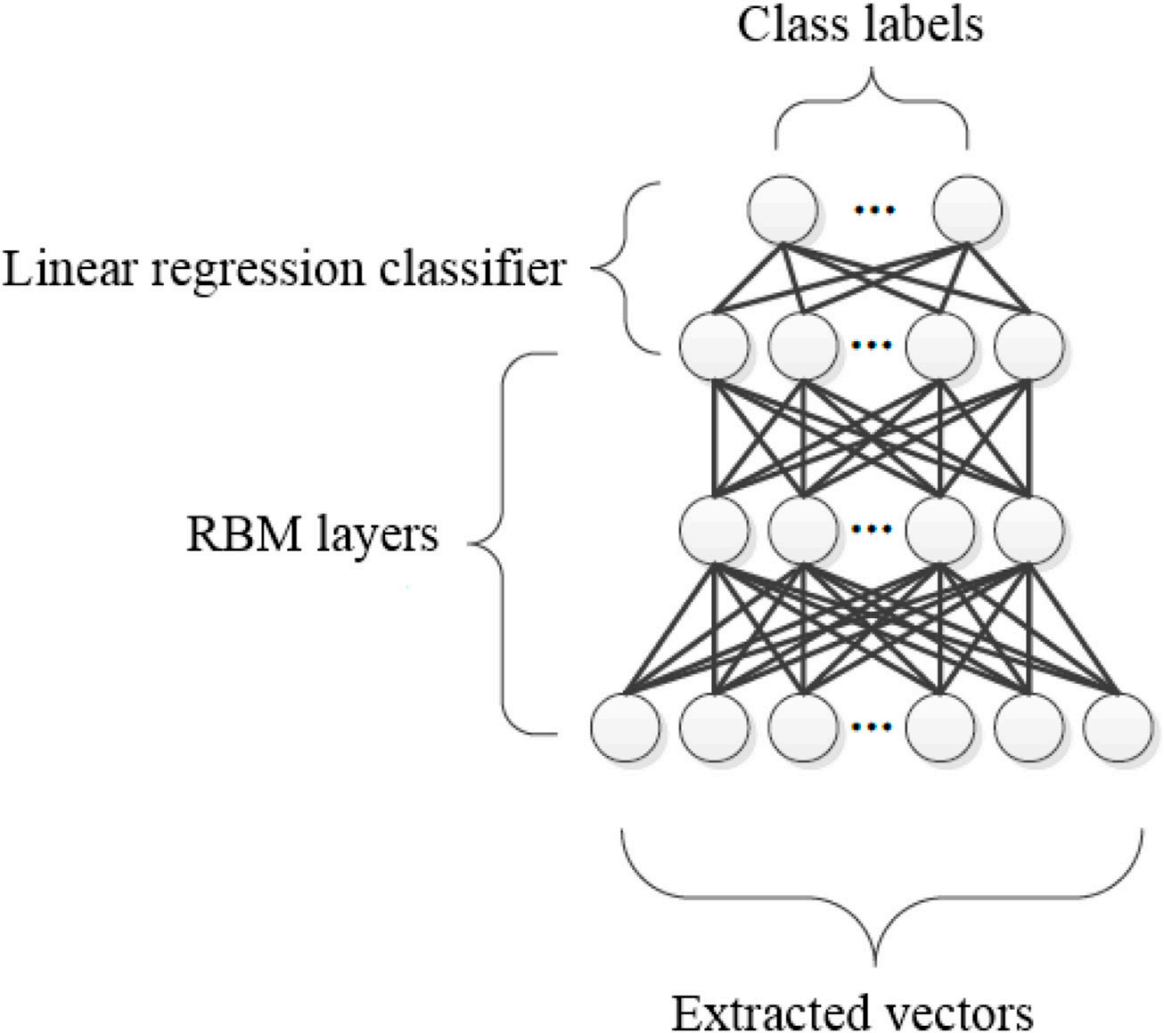
Schematic diagram of the DBN model.

The input of the DBN model are the two-dimensional vectors which are obtained during feature extraction. During pre-training, the RBM layers are trained with one another, and the visible values are considered as the duplicate of the hidden values in the prior layers. Here, the features are learned from the prior layers, and the learning parameters are transferred in a layer-wise manner. The linear regression is the final layer trained after fine-tuning. The cost function is updated by back propagation for optimizing the weights. The schematic diagram of the DBN model is shown in Figure 5.

By inspecting Figure 5, it is seen that the DBN model includes two major processes where: (i) RBM layers are trained unsupervised, and the inputs are mapped into dissimilar feature spaces, and (ii) linear regression layer is incorporated at the top of the DBN model as a supervised classifier. The parameters fixed in the DBN model are determined as follows: size of RBM layers is [8, 6], number of epochs is 100 for fine tuning, number of epochs is 10 for training RBM layers, batch size is 100 for training RBM layers, momentum is 0, learning rate is 0.01, activation function is sigmoid, and batch size is 100 for fine tuning. The numerical analysis of the Otsu-TSA method and DBN model is discussed in Section 4.

The performance of the proposed Otsu-TSA method and DBN model are analyzed using MATLAB R2022a software. The proposed framework is simulated on a system with 128GB RAM, NVIDIA GeForce RTX 3080 Ti graphics card, and Windows (64-bit) operating system. The Otsu-TSA method’s efficacy is investigated based on three different performance measures: JSC, DSC, and PA. In addition, the DBN’s effectiveness is validated on the basis of 4 performance measures, namely: specificity, classification accuracy, execution time, and sensitivity on the ADNI and AIBL datasets.

In the segmentation phase, the JSC estimates the degree of overlap between the masks or bounding boxes, while the DSC quantifies the similarity between the masks. The formulas to compute JSC and DSC are represented in Eqs 19, 20. The terms PS and GS are represented as the pixels in the predicted segmentation and ground-truth segmentation. Additionally, PA is determined as the ratio of the number of misclassified pixels in a class ${pixel}_{ii}$ to the total number of pixels in a class ${pixel}_{ij}$ . Where, the term K is denoted as class. The mathematical expression of PA is specified in Eq. 21.

$$JSC\left(PS,GS\right)=\frac{\left|PS\cap GS\right|}{\left|PS\cup GS\right|}$$ (19)

$$DSC\left(PS,GS\right)=\frac{2\times \left|PS\cap GS\right|}{\left|PS\right|+\left|GS\right|}$$ (20)

$$PA=\frac{\sum_{i=0}^{K}{pixel}_{ii}}{\sum_{i=0}^{K}\sum_{j=0}^{K}{pixel}_{ij}}$$ (21)

Correspondingly, in the classification phase, specificity estimates the proportion of TNs that are precisely classified by the DBN model. On the other hand, the sensitivity estimates how well the DBN model detects positive instances in AD detection. The classification accuracy is determined as the ratio of the number of correct predictions to the total input samples. In this scenario, the performance measures: specificity, accuracy, and sensitivity are estimated using the default threshold value of 0.5. The mathematical formulas of the undertaken performance measures are described in Eqs 22–24. Where, TP states true positive, TN states true negative, FP depicts false positive, and FN represents false negative.

$$Specificity=\frac{TN}{TN+FP}\times 100$$ (22)

$$Accuracy=\frac{TP+TN}{TP+TN+FP+FN}\times 100$$ (23)

$$Sensitivity=\frac{TP}{TP+FN}\times 100$$ (24)

## 4 Results

Here, the performance of the proposed segmentation method (Otsu-TSA) is compared with other segmentation methods like: region growing, K-means clustering, Watershed algorithm, superpixel clustering, and Otsu thresholding in terms of JSC, DSC, and PA on both ADNI and AIBL datasets which is clearly described in the following subsections.

### 4.1 Analysis related to region segmentation

**FIGURE 6:**
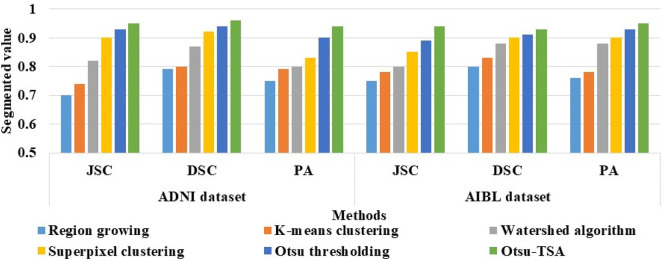
Visual evaluation of the Otsu-TSA method and the comparative segmentation methods.

The results of the Otsu-TSA method and the comparative methods are described in Table 3. By analyzing Table 3, it is clear that the proposed Otsu-TSA method achieves better segmentation than other segmentation methods. The proposed Otsu-TSA method achieved 0.95 of JSC, 0.96 of DSC, and 0.94 of PA on the ADNI dataset. The method achieved 0.94 of JSC, 0.93 of DSC, and 0.95 of PA on the AIBL dataset, which are far better than the results of the other segmentation methods. The conventional Otsu thresholding method uses an exhaustive search approach for region segmentation, which is computationally complex and consumes high processing time. Therefore, TSA is introduced in the conventional Otsu thresholding method to optimize the threshold value. The visual evaluation of the Otsu-TSA method and the comparative segmentation method’s results are shown in Figure 6.

**TABLE 3:** Achieved results of the Otsu-TSA method and the comparative segmentation methods.

Methods	ADNI dataset			AIBL dataset		
	JSC	DSC	PA	JSC	DSC	PA
Region growing	0.70	0.79	0.75	0.75	0.80	0.76
K-means clustering	0.74	0.80	0.79	0.78	0.83	0.78
Watershed algorithm	0.82	0.87	0.80	0.80	0.88	0.88
Superpixel clustering	0.90	0.92	0.83	0.85	0.90	0.90
Otsu thresholding	0.93	0.94	0.90	0.89	0.91	0.93
Otsu-TSA	0.95	0.96	0.94	0.94	0.93	0.95

### 4.2 Analysis related to classification

The effectiveness of the DBN model is validated by comparing its results with other classification models (Autoencoder, LSTM, GRU, AlexNet, and DenseNet) on the ADNI and AIBL datasets. These five comparative models are also executed on the same environment and system configuration. The parameter settings of the comparative models for AD detection are presented as follows. Specifically, the parameters considered in the autoencoder model are as follows: learning rate is 0.001, batch size is 128, optimizer is Adam, dropout rate is 0.15, number of hidden layers is 5, number of nodes in every layer is 500, epochs are 100, and activation is ReLU. The common parameters assumed in the LSTM and GRU models are presented the following: cost function is cross entropy, activation functions are sigmoid and tangent, minimum batch size is 64, epochs is 100, and learning rate is 0.001. Further, the common parameters considered in the AlexNet and DenseNet models are set as follows: epochs are 100, train batch size is 128, dropout rate is 0.5, momentum is 0.9, and weight decay is 0.005.

**FIGURE 7:**
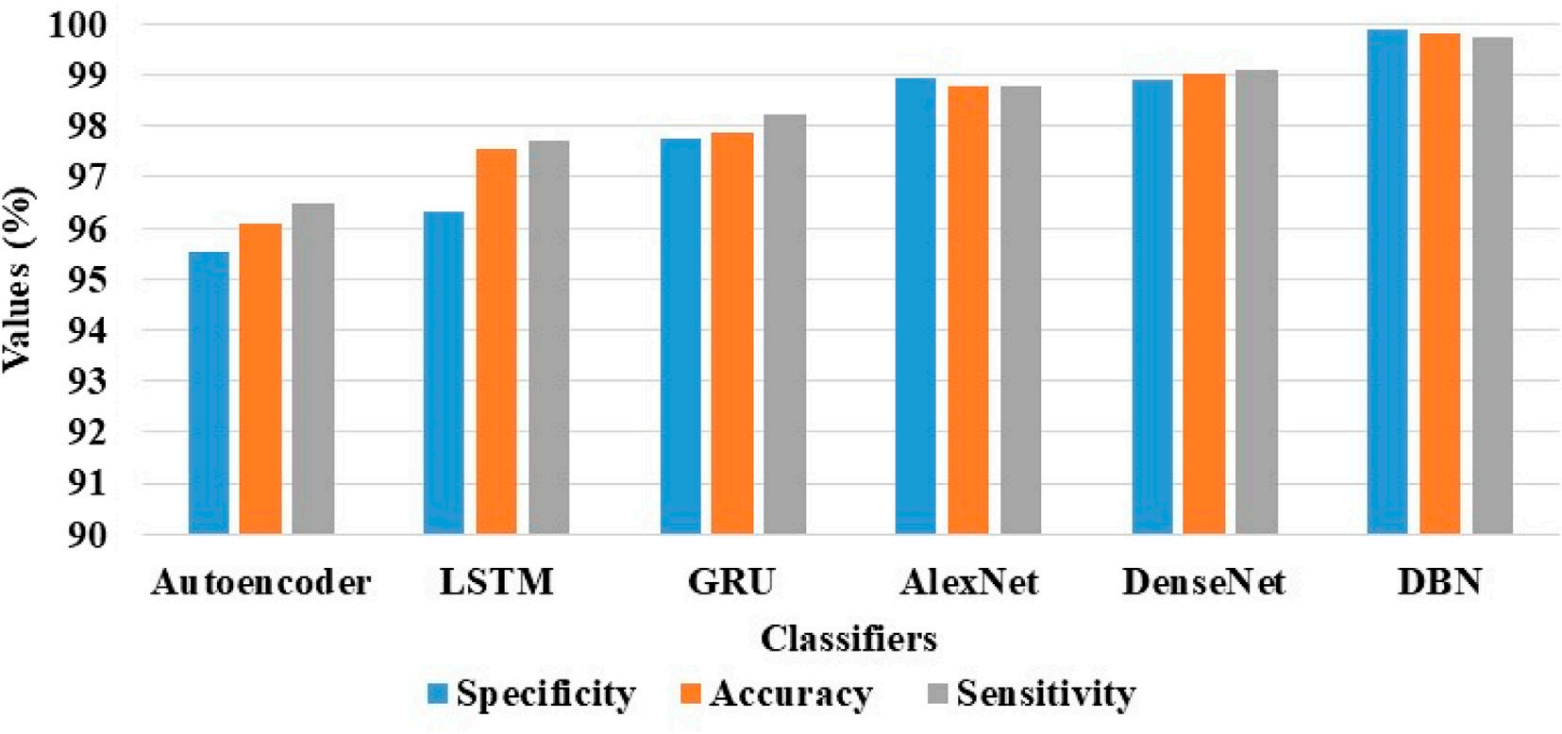
Visual evaluation of the DBN and the comparative classification models results on the ADNI dataset.

The results of the DBN model and the comparative classification models on the ADNI and AIBL datasets are described in Tables 4, 5. As mentioned in Table 4, the DBN model’s efficacy is compared with 5 different classification models: autoencoder, Long Short Term Memory (LSTM) network, Gated Recurrent Unit (GRU), AlexNet, and DenseNet. In the ADNI dataset, the DBN model achieves supreme results with a specificity of 99.88%, classification accuracy of 99.80%, and sensitivity of 99.72%. The visual evaluation of the performances of DBN and the comparative classification models on the ADNI dataset are shown in Figure 7. In this scenario, the DBN is able to handle more feature vectors than the comparative classification models. Due to its usage and robustness of the hidden layers, the DBN assembles useful correlations of the feature vectors to achieve better image classification.

**TABLE 4:** Achieved results of the DBN model and the comparative classification models on the ADNI dataset.

ADNI dataset			
Classifiers	Specificity (%)	Accuracy (%)	Sensitivity (%)
Autoencoder	95.52	96.10	96.48
LSTM	96.32	97.56	97.72
GRU	97.77	97.86	98.22
AlexNet	98.93	98.78	98.80
DenseNet	98.92	99.02	99.10
DBN	99.88	99.80	99.72

**TABLE 5:** Achieved results of the DBN model and the existing classification models on the AIBL dataset.

AIBL dataset			
Classifiers	Specificity (%)	Accuracy (%)	Sensitivity (%)
Autoencoder	97.58	97.83	97.98
LSTM	97.65	98.82	98.90
GRU	98.60	98.80	99.12
AlexNet	98.90	99.04	99.30
DenseNet	99.22	99.20	99.33
DBN	99.82	99.92	99.78

**FIGURE 8:**
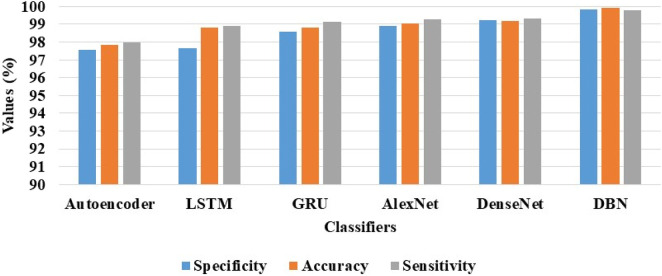
Visual evaluation of the DBN and the comparative classification models results on the AIBL dataset.

Correspondingly, in the AIBL dataset, the DBN model achieves supreme classification results with 99.82% of specificity, 99.92% of classification accuracy, and 99.78% of sensitivity, as mentioned in Table 5. Figure 8 is the visual evaluation of DBN and the comparative classification models’ results on the AIBL dataset.

### 4.3 Comparative analysis

The effectiveness of the proposed framework is validated by comparing its results with the existing model developed by Yue et al. (2019) on the ADNI dataset. Initially, an automated anatomical labelling is performed to segment RoI from the acquired sMRI images. Then, the informative voxels are extracted in the segmented regions, which are finally fed to the CNN model for image classification (NC, MCI, and AD). The experimental outcomes of the proposed framework and the existing model developed by Yue et al. (2019) are described in Table 6. Here, the experiment was conducted for 1662 ADNI sMRI images.

**TABLE 6:** Comparative outcomes on the ADNI dataset.

Classification accuracy (%)			
ADNI dataset	NC vs. MCI	MCI vs. AD	AD vs. NC
CNN Yue et al. (2019)	98.90	97.80	99.70
DBN	99.88	99.81	99.71

On the other hand, the proposed framework’s efficacy is compared with the existing model developed by Alhassan (2022) on the AIBL dataset. In the existing study, the sMRI image denoising is initially carried out utilizing linear registration, skull stripping, and geometry correction techniques. From the denoised sMRI images, the RoI segmentation is performed by combining the EFEHO algorithm with the Otsu thresholding method, and finally, the image classification is done by implementing the DA-MIDL model. By examining the experimental results, the DA-MIDL model obtained 86.50% of accuracy, 93% of sensitivity, and specificity on the AIBL dataset with 320 sMRI images. Whereas, the DBN model obtained 99.92% of classification accuracy, 99.82% of specificity, and 99.78% of sensitivity on the AIBL dataset with 320 sMRI images, which is described in Table 7. The benefits of proposing the Otsu-TSA method and the DBN model in AD detection are briefly detailed in the discussion section.

**TABLE 7:** Comparative outcomes on the AIBL dataset.

AIBL dataset	Accuracy (%)	Sensitivity (%)	Specificity (%)
DA-MIDL Alhassan (2022)	86.50	93	93
DBN	99.92	99.78	99.82

### 4.4 Discussion

In AD detection, the proposed framework comprises of two important steps, which are: region segmentation carried out by the Otsu-TSA method, and the AD classification carried out by the DBN model. After the acquisition of sMRI images, the RoI is segmented by integrating the Otsu thresholding method with TSA. The TSA finds the optimal threshold value in the Otsu thresholding method for fast and precise segmentation, and the selection of optimal threshold value reduces the execution time of the proposed framework. In the segmented RoI, the vectors are extracted by implementing LDPv and LBP descriptors. The conversion of images into vectors boosts the learning rate, decreases redundant data, and improves the accuracy of the classification model. The extracted vectors are passed as the input to the DBN model for categorizing three classes (NC, MCI, and AD) in the ADNI dataset, and four classes (NC, AD, sMCI and pMCI) in the AIBL dataset. The DBN has high generalization and learning ability than the DenseNet model on the sMRI images, therefore, it is effective in obtaining maximum classification results in AD detection. The execution time of different classification models on the ADNI and AIBL datasets is described in Table 8, and it also shows the effectiveness of incorporating TSA in the Otsu thresholding method.

**TABLE 8:** Execution time of different classification models on the ADNI and AIBL datasets.

Execution time (seconds)		
Classifiers	ADNI dataset	AIBL dataset
Autoencoder	112.02	103.12
LSTM	78.34	66.10
GRU	74.33	63.29
AlexNet	55.10	44.54
DenseNet	53.44	40.14
DBN	48.58	36.38

## 5 Conclusion

In this paper, an effective AD detection framework was proposed based on the Otsu-TSA method and DBN model. The proposed AD detection framework included three steps: RoI segmentation using the Otsu-TSA method, extraction of vectors using hybrid descriptors (LDPv and LBP) and image classification by the DBN model. The performance of the proposed segmentation method (Otsu-TSA) and classification model (DBN) were analyzed on 1662 ADNI subjects with 3 classes and 496 AIBL subjects with 4 classes. The performance measures: JSC, DSC, PA, specificity, classification accuracy, and sensitivity were used for evaluating the effectiveness of the proposed segmentation method and classification model. In comparison with the existing methods, the proposed Otsu-TSA method achieved higher PA of 0.94 and 0.95 on the ADNI and AIBL datasets, respectively. Additionally, the classification model DBN obtained maximum accuracy of 99.80% and 99.92% on the ADNI and AIBL datasets, which is superior to the conventional detection models like autoencoder, LSTM, GRU, AlexNet, and DenseNet. The results demonstrated that the proposed framework not only achieved better diagnosis performance, but also precisely identified the discriminative pathological locations in the sMRI images than the comparative systems. The selection of optimal threshold value in the Otsu thresholding method by TSA reduced the execution time of the proposed framework to 48.58 and 36.38 s on the ADNI and AIBL datasets.

However, the integration of an optimization algorithm in the segmentation method increases the computational complexity that needs to be overcome in the future. As a future extension, a novel unsupervised model can be developed with a grid search hyper-parameter optimization method for diminishing the computational complexity, and can be applied in other diseases like lung cancer and breast cancer detection.

## Data Availability

The datasets generated during and/or analysed during the current study are available in the ADNI and AIBL datasets repository, https://adni.loni.usc.edu/data-samples/access-data/ and https://aibl.csiro.au/adni/index.html.
